# Night shifts in interns: Effects of daytime napping on autonomic activity and cognitive function

**DOI:** 10.3389/fpubh.2022.922716

**Published:** 2022-10-10

**Authors:** Jieyi Fan, Liang Wang, Xiaotian Yang, Xiangbo Zhang, Ziyao Song, Sifan Wu, Linru Zou, Xi Li, Xingcheng Zhao, Chenfei Li, Yikai Pan, Yateng Tie, Yongchun Wang, Zhengxue Luo, Xiqing Sun

**Affiliations:** ^1^Department of Aerospace Medicine, Air Force Medical University, Xi'an, China; ^2^Department of Medical Genetics and Developmental Biology, Air Force Medical University, Xi'an, China; ^3^General Hospital of PLA Air Force, Beijing, China

**Keywords:** night shifts, daytime napping, autonomic activity, cognitive function, intern

## Abstract

**Objective:**

Night shifts have adverse cognitive outcomes that might be attenuated by daytime napping. The neurovisceral integration model suggests that resting vagally mediated heart rate variability (vmHRV) is linked with cognitive function. This study investigated the relationship between resting vmHRV and cognitive function after different nap durations in interns after shift work.

**Methods:**

A total of 105 interns were randomly allocated to one of three groups (non-nap, *n* = 35; 15-min nap, *n* = 35; 45-min nap, *n* = 35) to perform cognitive tests and resting vmHRV at 12:00, 15:00 and 18:00. Information processing (digit symbol substitution test; DSST), motor speed (finger tapping test; FTT), response selection (choice reaction time; CRT), and attention shifts (shifting attention test; SAT) were assessed. Resting vmHRV was assessed at baseline and during each cognitive task across groups.

**Results:**

Compared with the non-nap control, the 15-min and 45-min naps improved all outcome measures (including subjective sleepiness and cognitive performance) at 15:00, with some benefits maintained at 18:00. The 15-min nap produced significantly greater benefits on the FTT at 15:00 after napping than did the 45-min nap. Resting vmHRV was significantly correlated with DSST and SAT performance. In addition, FTT performance was the only significant predictor of DSST performance across different nap durations.

**Conclusion:**

Our results demonstrate links between daytime napping (in particular, a 15-min nap) and improved cognitive control in relation to autonomic activity after shift work in interns. These results indicated that autonomic activity when awake plays a crucial role in DSST and SAT performance and facilitated the understanding of differences in neurocognitive mechanisms underlying information processing after different nap durations.

## Introduction

In China, the population of shift workers is approximately 80 million (1). With the busy work schedule and the increasingly tense work environment, Chinese healthcare shift workers are facing tremendous mental pressure and risk. Shift work involving circadian disruption may disturb the sleep/awake cycle. Altered sleep/wake rhythms of interns working night shifts has been associated with decreased vigilance and psychological stress (2), which is not only associated with various negative health outcomes such as obesity, cardiovascular disease, and cancers (2–9), but also potentially related to medical errors and adverse patient-related outcomes (2, 10–13). Schernhammer ES (8) found that night shifts improved the risk of colorectal cancer in the nurses. Other research also showed that night shifts were the important risk factor for breast cancer (9). Medical errors among healthcare shift workers during night shifts is well documented. Trauma residents made more cognitive errors using a simulated laparoscopic exercise due to sleep loss (12). Rothschild JM also observed that increasing rates of nighttime surgery complications were performed by attending physicians who had slept less than 6 h the previous night (13). Night shift work can lead to alterations in cognition, such as alterations in alertness, reaction times, and information processing (14).

Regarding psychological health and performance, one important cognitive process is information processing measured by the digit symbol substitution test (DSST). The DSST has been considered a “direct measure of the rate of information processing of visual figures” (15). Research has consistently observed the relationship between sleep, DSST performance, and sleep disorders, such that worse sleep quality and cognitive decline among older adults and long sleep durations (>9 h) were associated with 7-year neurocognitive decline in middle-aged to older adults (16). There are different interrelated components involved in and considered critical for good DSST performance: motor speed, relational selection, and attentional shift. To identify and assess different cognitive components that contribute to performance in the DSST, we isolated these cognitive components using a cognitive test battery that included the finger tapping test (FTT), choice reaction time (CRT), and shifting attention test (SAT) to measure motor speed, response selection, and attentional shift, respectively.

It is crucial to provide strategies that attenuate the adverse effects of shift work to ensure medical professionals' safety and health, especially their cognitive performance. Napping prior to a night shift or during the work shift has been shown to improve alertness and performance and decrease accident rates (17, 18). During shift work, naps of 20 to 50 min in duration have been associated with improvements in reaction time and restoration of performance to that seen at the start of the shift. Napping early in the night shift can improve objective measures of alertness (17). To avoid sleep inertia that sometimes occurs when waking from a nap, naps should not be longer than 50 min and can be as short as 10 to 15 min (17, 19). Naps as brief as 15 and 20 min have been shown to improve alertness following normal nocturnal sleep (20, 21). Brief naps during the night shift have also been observed to be beneficial (17). A comparison between two nap opportunities (15 vs. 45 min) following normal sleep was examined in the current study. Takahashi et al. (22) showed significantly improved alertness 30 min after the 15-min nap and comparable improvements for the two nap conditions 3 h after napping. Tietzel and Lack (23) found that 10-min nap in the afternoon was at least as recuperative as a 30-min nap in terms of improved alertness and cognitive performance for an hour following the napping after restricted nocturnal sleep. While longer naps (1 to 2 h) have been shown to be more restorative than brief naps for at least 3 h after napping (24, 25) following a night of total sleep loss. However, it is important to find the limitations of these studies. Previous studies measured the effects of different nap conditions during the 3-h time course even only 1-h time course. There is clearly a need to examine the effects of different nap conditions over an extended postnap period. Therefore, the purpose of our study was to examine the effects of different nap durations on performance after nap during long time course (i.e., 6 h). The study arranged tests at 15:00 and 18:00 after nap. Additionally, a comparison between these two nap durations (15 vs. 45 min) was examined in our study. These two durations (15 vs. 45 min) of nap opportunities were chosen in our study because, based on previous studies, a comparison between 15 and 45-min nap durations was only examined following normal sleep (22). Considering that following restricted sleep among interns, the benefits of nap durations must be weighted against their practical disadvantages. The practical aim of our study was to provide information that would allow a more informed decision regarding the most effective nap duration for improving cognitive performance in interns.

Research has shown that resting vagally mediated heart rate variability (vmHRV) may predict performance in a cognitive task (26). Some theoretical models have provided a foundation on a number of conceptual approaches linking autonomic activity and cognitive function. The polyvagal theory emphasizes the importance of the vagus nerve in regulating social behaviors and suggests that high resting vmHRV is associated with improved emotion perception (27). Another relevant model is the neurovisceral integration model (28) that indicates HRV as an essential index of adaptability and emphasizes the relationship between cognitive function and HRV (29). HRV has an effect on cognitive performance because of its ability to influence activity in prefrontal neural structures (30, 31), especially the high frequency (HF) component of the HRV, which is an index of parasympathetic control and vagal tone (32). According to the neurovisceral integration model, vmHRV plays an important role as an indicator of functional activity in the prefrontal cortex, a key area that drives cognitive control, as the heart and brain are connected *via* the vagus nerve. HRV is a non-invasive measure of beat-to-beat temporal changes in heart rate (27). vmHRV is an important index of neurovisceral integration and organismic self-regulation (33). Evidence supports the notion that resting vmHRV not only contributes to the parasympathetic nervous system and cardiac regulation but also is associated with the brain's integrative system for self-regulation in cognitive tasks (26, 28, 34, 35). Further investigation that vagal activity is associated with different cognitive tasks in the extent of different nap durations is warranted.

We investigated whether different nap durations have effects on cognitive function and whether this relationship is associated with resting vmHRV. We examined cognitive function, including information processing, motor speed, response selection, and attention shifts, measured by the DSST, FTT, CRT, and SAT in interns. In particular, the study aimed (1) to compare the differential effects of not napping, a 15-min nap, and a 45-min nap on cognitive performance, (2) to assess resting vmHRV following different nap durations during cognitive task performance, and (3) to explore the different cognitive components that contribute to performance in the DSST. We hypothesized that daytime napping, especially a 15-min nap, would benefit cognitive performance to a greater extent than non-nap conditions and that the variability in resting vmHRV during cognitive tasks would depend on the particular cognitive component and the effect of nap conditions.

## Materials and methods

### Participants

A total of 108 interns from the Air Force Medical University First Affiliated Hospital were recruited as volunteers. On-call interns were responsible for admitting patients throughout the night until the primary team returned at approximately 7 am and generally worked until approximately 12 pm the next day. During the night shifts, interns assist the superior doctor to deal with medical emergency, such as shock, pain, bleeding, and other medical problems. The mainly work of interns is responsible for learning treatment strategies and procedures, which needs cognitive demands, such as attention, vigilance, and information processing. They were healthy, did not have a history of psychiatric or medical disorders, and did not smoke or drink caffeine during the experiment. The interns were informed about all potential risks of the study and were supervised through the study. The participants provided written informed consent. The study was approved by the ethical committee of the Air Force Medical University.

### Questionnaire

Before entering the protocol, interns completed questionnaires about their sleep status and daily habits. These questionnaires were the Pittsburgh Sleep Quality Index (PSQI) (36) and the morningness–eveningness questionnaire (37). The PSQI provided an index of the average sleep quality over the last month. Individuals with scores >5, indicating poor sleep quality, were excluded from the experiment. The morningness–eveningness questionnaire was designed to determine the circadian chronotype. Extreme morning (>70) and extreme evening (<31) chronotypes were excluded from the experiment.

### Design and procedure

Out of 108 possible permutations of order for 3 conditions, 36 orders were selected at random. Interns were randomly arranged an order such that each nap condition happened an approximately equal number of times in each position order.

#### Assessment of subjective ratings

The participants completed the Stanford Sleepiness Scale (SSS) for the assessment of subjective sleepiness (38). The scale ranged from “1” as “very alert” to “7” as “very sleepy”. Based on the aim of the study, the participants completed the self-rated sleepiness scale before the cognitive tests at 12:00, 15:00, and 18:00.

#### Cognitive performance

All tests were administered to the 15-min and 45-min groups before and after napping. The cognitive tests includes the DSST, FTT, CRT, and SAT. Motor speed measured by the FTT, response selection measured by the CRT, shifting of attention measured by the SAT, and information processing measured by the DSST were the four cognitive components. Table 1 provides a detailed presentation of the cognitive tests.

**Table 1:** Description of different cognitive tests.

**Tests**	**Cognitive components**	**Details of the tests**	**Duration of test**	**References**
Digit Symbol Substitution Test (DSST)	Motor speed, relational selection, attentional shift, working memory, and visual scanning efficiency	Each item in the parametric DSST included the presentation of a reference set of digit symbol pairs in the upper row at the top of each screen. A series of numbers from 1 to 9 corresponded to different symbols. At the middle of the screen, the symbols were presented in random order. Subjects were required to indicate a reference set of digit symbol pairs in the upper row *via* digit button press. DSST was performed for 3 min.	90s	(39)
Finger Tapping Test (FTT)	Motor speed	Subjects were instructed to watch the screen. Each subject selected his or her most flexible finger for the repeated tapping of the button for 10 s until a red light appeared. The test was repeated six times. The first three times were done on the right hand and next three on the left hand.	10s	(40)
Choice Reaction Time (CRT)	Selection response	Three stimuli of red, yellow, and green plots were randomly presented, and the test–retest reliability for the accurate rate in CRT was r = 0.80.The CRT required 3 min to complete.	90s	(41)
Shifting Attention Test (SAT)	Attentional shift	SAT was involved in one reference set in the first row and two different figures in the second row. The subjects were instructed to match the geometric objects either by shape or color. Three figures were presented on the screen, such that one reference was set on top and two different figures at the bottom. The top reference set was either a square or a circle. The bottom figures were square and circle. All figures were either red or blue (mixed randomly). Participants were instructed to match one of the bottom figures to the top reference set. Figures were matched by shape or color randomly. The SAT took 90 s to complete.	90s	(42–44)

#### Electroencephalography recording

EEG was collected and analyzed using Scan 4.3 from a 32-channel array (10/20 system) with an Ag–AgCl electrode cap (NeuroScan Inc., Compumedics, Australia). EEG was collected from channels C3, C4, F3, F4, O1, and O2, and A1 and A2 were used as references. Bipolar horizontal and vertical electrooculography (EOG) electrodes placed 1 cm from the outer canthus of each eye were recorded at a 500-Hz sampling rate, and electromyography (EMG) was recorded *via* a bipolar channel. The skin-electrode impedance was maintained below 5 kΩ. A 50-Hz notch filter was used for the recording with bandpass filtering ranging from 0.1 Hz to 100 Hz. If the participants obtained slow-wave sleep (SWS) during the 15-min or 45-min nap, then they were allowed to continue sleeping until the SWS period ended. The participants were awakened only from sleep stage 1 or 2. The participants who entered rapid eye movement (REM) were excluded from the data analysis. The sleep recordings were scored using the 1968 International Criteria of Rechtschaffen and Kales (45).

#### Physiological measures

Psychophysiological data were collected using a three-electrode differential biological amplifier (KF2 dynamic multi-parameter physiological detector, Beijing Baomai Technology Company, China). R-waves series were automatically detected by KF2 dynamic multi-parameter physiological detector software and visually examined by skilled technicians. Incorrectly detected R-peaks were manually edited. Ectopic beats were corrected using the automatic medium filter provided by KF2 dynamic multi-parameter physiological detector software. Frequency domain measures of HRV were calculated using the classical power spectral estimation method. To remove abnormal RR intervals and obtain RR1 intervals, three-spline interpolations were performed. A sampling rate of 2 Hz was applied for the recording, and an RR2 interval was obtained. Frequency domain measures were obtained by performing fast Fourier transform. Analysis of the power spectra was performed on low frequency (LF: 0.04–0.15 Hz) and high frequency (HF: 0.15–0.40 Hz) heartbeats. The LF and HF measures were expressed in normalized units (LFnu and HFnu). LFnu and HFnu are ratio indexes that eliminate interference factors from LF and HF and reflect the relative activity of the sympathetic nervous system and parasympathetic nervous system. The LFHRV and HFHRV measures had skewed distributions and were transformed by taking the natural logarithm (46). From these variables, we derived the HF normalized units (HFnu = (HFHRV [ms^2^]/HFHRV [ms^2^] + LF HRV [ms^2^]) × 100). Because the LF normalized units are mathematically reciprocal to HFnu, we computed only the HFnu index, which is often thought to reflect vagal modulation.

#### Procedure

**Figure 1:**
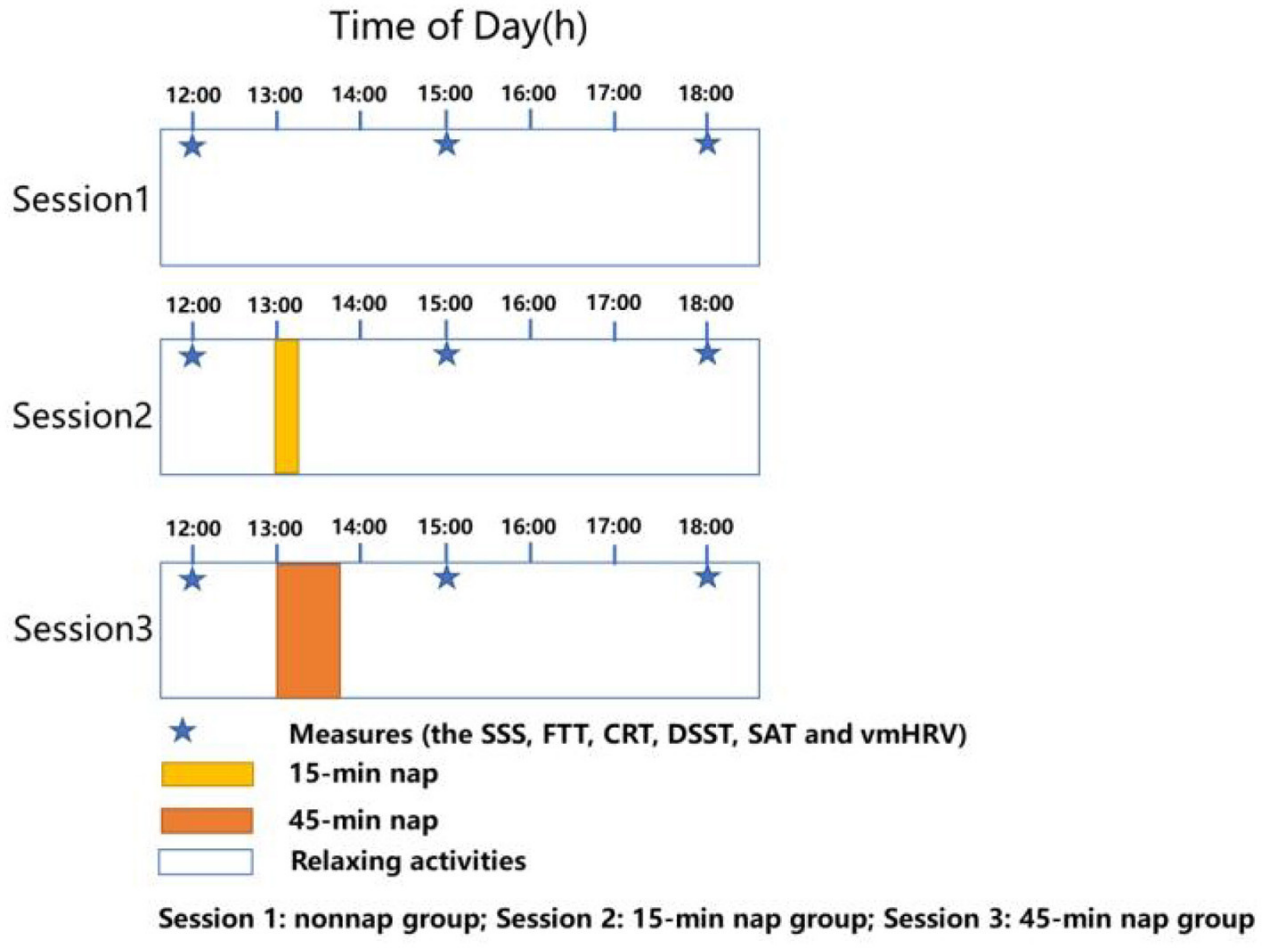
Protocol design.

Figure 1 shows that our study consisted of three sessions (session 1, session 2, and session 3). In session 1, the participants were instructed to take a 15-min nap. In session 2, the participants were required to take a 45-min nap. In session 3, the participants did not nap.

Before the study, the participants were informed that the cognitive tests would be conducted five times to reduce practice effects. The participants completed the SSS and cognitive tests at 12:00. Before the baseline cognitive tests, the participants were instructed to sit quietly for 2 min, during which time, their “resting” heart rates were obtained. Thereafter, the participants underwent baseline cognitive testing with heart rate monitoring. They were then randomly assigned to the non-nap group (*n* = 35), 15-min nap group (*n* = 35) or 45-min nap group (*n* = 35). At 13:00, the participants in the 15-min nap and 45-min nap groups were taken to two sound-attenuated bedrooms for the naps. The two nap groups were required to sleep for up to 15 and 45 min. EEG was recorded throughout both nap conditions to monitor sleep.

At the end of the nap period, all the participants exited their respective bedrooms, and the electrodes were removed. They then watched videos until the testing session. At 15:00 and 18:00, all the participants repeated the above baseline assessments and cognitive tests with heart rate monitoring.

At 12:00, the participants completed a battery of tests (i.e., the SSS, FTT, CRT, DSST, and SAT). Prior to the baseline tests, the participants were assessed vmHRV in the rest condition was calculated. Afterwards, they performed the baseline cognitive tests with vmHRV monitoring throughout. The participants were assigned to the non-nap group, 15-min nap group, or 45-min nap group. At 13:00, the participants had an uninterrupted nap (15-min and 45-min nap) or remained awake with access to movies. EEG was recorded throughout the 15-min nap and 45-min nap. At the end of the nap, the electrodes were removed. After a period of free time, they were retested with the above baseline assessments and cognitive tests with vmHRV monitoring at 15:00 and 18:00.

### Statistical analysis

IBM SPSS Statistics (version 21.0, SPSS, Inc., an IBM company, Chicago, IL, USA) was used for all analyses. Sleep data from the 15-min nap and 45-min nap groups were analyzed using descriptive statistics. Statistical analyses were carried out for each variable (i.e., the SSS, DSST, SAT, FTT, CRT, and HRV).

For all variables, 2-way repeated-measures ANOVA comprising 3 levels on the factor nap length (non-nap, 15-min nap, and 45-min nap) and 3 levels on the factor time (12:00, 15:00 and 18:00) was performed. For cognitive performance and HRV variables, simple within-subjects planned contrasts were then performed. The overall main differences between nap groups and times were not relevant to testing the aims of this study. The interest was in the relative changes from before to after nap between the non-nap group and other nap lengths, as investigated by examining the interaction effects between nap lengths and testing time. Factors influencing performance on the DSST performance were assessed by means of linear or non-linear hierarchical regression, as appropriate. To confirm that there were no differences in cognitive performance at 12:00 (baseline) among the three nap groups, we used one-way ANOVA with nap condition (non-nap, 15-min nap, and 45-min nap) as the between-participant factor and cognitive performance at 12:00 as the dependent variable. To assess the relative importance of HRV variables for cognitive performance, we utilized a hierarchical linear regression approach. In Model 1, cognitive performance was the independent variable, and test session was the dependent variable. In Model 2, we added the HRV factors as independent variables. By comparing Models 1 and 2, we measured the explanatory gain of HRV factors over and above individual differences in cognitive performance. All comparisons were adjusted by Bonferroni correction. Tests were calculated with an alpha of 0.05.

## Results

Out of the original 108 interns, data from 3 were excluded. Two interns in 15-min nap group did not enter Stage 1 (S1), Stage 2 (S2) or SWS; while one intern terminated heart rate monitoring at 15:00. 105 interns (23–25 years old; mean ± standard error of mean: 24.3 ± 0.4) were included in final analyses. These interns were either in the non-nap group (*n* = 35; 35 males; mean ± SD: 24.1 ± 0.4 years), 15 min nap group (*N* = 35; 35 males; mean ± SD: 24.6 ± 0.3 years) or 45 min nap group (*n* = 35; 35 males; mean ± SD: 24.2 ± 0.4 years). EEG was recorded throughout both nap conditions to monitor sleep.

### Sleep data

The participants who napped achieved physiological sleep during the 15-min and 45-min nap opportunities, as defined by the 1968 International Criteria of Rechtschaffen and Kales (45). In the 15-min nap group, the participants slept an average of 13.96 ± 1.21 min (mean ± SD), with 2.85 ± 1.01 min in stage 1 (S1), 7.26 ± 3.14 min in stage 2 (S2), and 1.98 ± 1.24 min in SWS. In the 45-min nap group, the participants slept an average of 43.18 ± 2.12 min, with 8.56 ± 2.33 min in stage 1 (S1), 22.64 ± 6.55 min in stage 2 (S2), and 5.85 ± 2.07 min in SWS.

#### Assessment of subjective ratings

In the non-nap group, the participants showed a high level of sleepiness indicated by a significant increase in their SSS scores after a main effect “nap condition” was obtained [*F*_(2, 101)_ = 5.10, *p* = 0.02]. A significant main effect of “time of day” [*F*_(2, 101)_ = 6.89, *p* < 0.01] was observed. No significant “nap condition × time of day” interactions were observed [*F*_(2, 101)_ = 1.13, *p* = 0.34). In the 15-min nap group, the main effects of “nap condition” [*F*_(2, 101)_ = 4.59, *p* = 0.03] and “time of day” [*F*_(2, 101)_ = 6.13, *p* < 0.01] were also observed with lower SSS scores after napping, suggesting a high level of alertness. The “nap condition × time of day” interaction was not significant [*F*_(2, 101)_ =1.08, *p* = 0.38]. However, in the 45-min nap group, no significant differences were found as the main effects of “nap condition” [*F*_(2, 101)_ = 1.01, *p* = 0.06] and the “time of day” [*F*_(2, 101)_ = 1.63, *p* = 0.08] were not significant, and the “nap condition × time of day” interaction was not significant [*F*_(2, 101)_ =1.12, *p* = 0.36].

#### Cognitive performance and resting vmHRV

No significant differences were found in cognitive performance and resting vmHRV (12:00 baseline) among the 15-min nap, 45-min nap, and nonnap groups [FTT: *F*_(2, 101)_ = 0.24, *p* = 0.79; CRT: *F*_(2, 101)_ = 0.66, *p* = 0.52; SAT: *F*_(2, 101)_ = 0.15, *p* = 0.87; DSST: *F*_(2, 101)_ = 0.73, *p* = 0.49; HFnu (FTT): *F*_(2, 101)_ = 0.24, *p* = 0.79; HFnu (CRT): *F*_(2, 101)_ = 2.61, *p* = 0.08; HFnu (SAT): *F*_(2, 101)_ = 0.27, *p* = 0.76; HFnu (DSST): *F*_(2, 101)_ = 2.33, *p* = 0.11]. The results of 2-way interactions (nap condition × time of day) and simple planned contrasts against the non-nap group for all outcome measures are presented in Table 2. For the 15-min nap group, a significant planned contrast of interaction effects with the non-nap group was found with better performance for all cognitive outcome variables at 15:00 and better performance of FTT and CRT at 18:00 after the 15-min nap. In the 45-min nap group, the results also showed better performance for all cognitive outcome variables at 15:00 and better performance of FTT and CRT at 18:00 after the 45-min nap.

**Table 2:** Two-way ANOVA interaction effects and planned within-subjects contrasts for condition (non-nap vs. other nap condition) by time (pre-nap vs. post-nap time) for all dependent variables.

**Variable**	**ANOVA**	**Condition**	**Planned contrasts**	
			**Time**	
	**F**		**F**	
			**12:00 (baseline)** **vs. 15:00**	**12:00 (baseline)** **vs. 18:00**
FTT	13.97**	Non-nap vs 15-min nap Non-nap vs 45-min nap	42.73*** 41.36***	9.33** 6.27*
CRT	17.96**	Non-nap vs 15-min nap Non-nap vs 45-min nap	50.89*** 47.41***	30.62*** 16.04***
SAT	3.62**	Non-nap vs 15-min nap Non-nap vs 45-min nap	16.25*** 10.25**	3.00 1.16
DSST	2.88*	Non-nap vs 15-min nap Non-nap vs 45-min nap	5.33* 4.46*	0.01 0.09
HFnu(FTT)	2.68*	Non-nap vs 15-min nap Non-nap vs 45-min nap	8.97** 1.30	1.24 2.05
HFnu(CRT)	17.96**	Non-nap vs 15-min nap Non-nap vs 45-min nap	0.001 0.15	0.20 1.77
HFnu(SAT)	5.57**	Non-nap vs 15-min nap Non-nap vs 45-min nap	8.02** 2.62	0.57 0.48
HFnu(DSST)	3.98**	Nonnap vs 15-min nap Non-nap vs 45-min nap	15.12*** 10.76**	0.35 0.01

ANOVA refers to analysis of variance. FTT, Finger Tapping Test; CRT, Choice Reaction Time; DSST, Digit Symbol Substitution Test; and SAT, Shifting Attention Test; HFnu, high-frequency normalized units. * *p* < 0.05, ** *p* < 0.01, *** *p* < 0.001.

**Figure 2:**
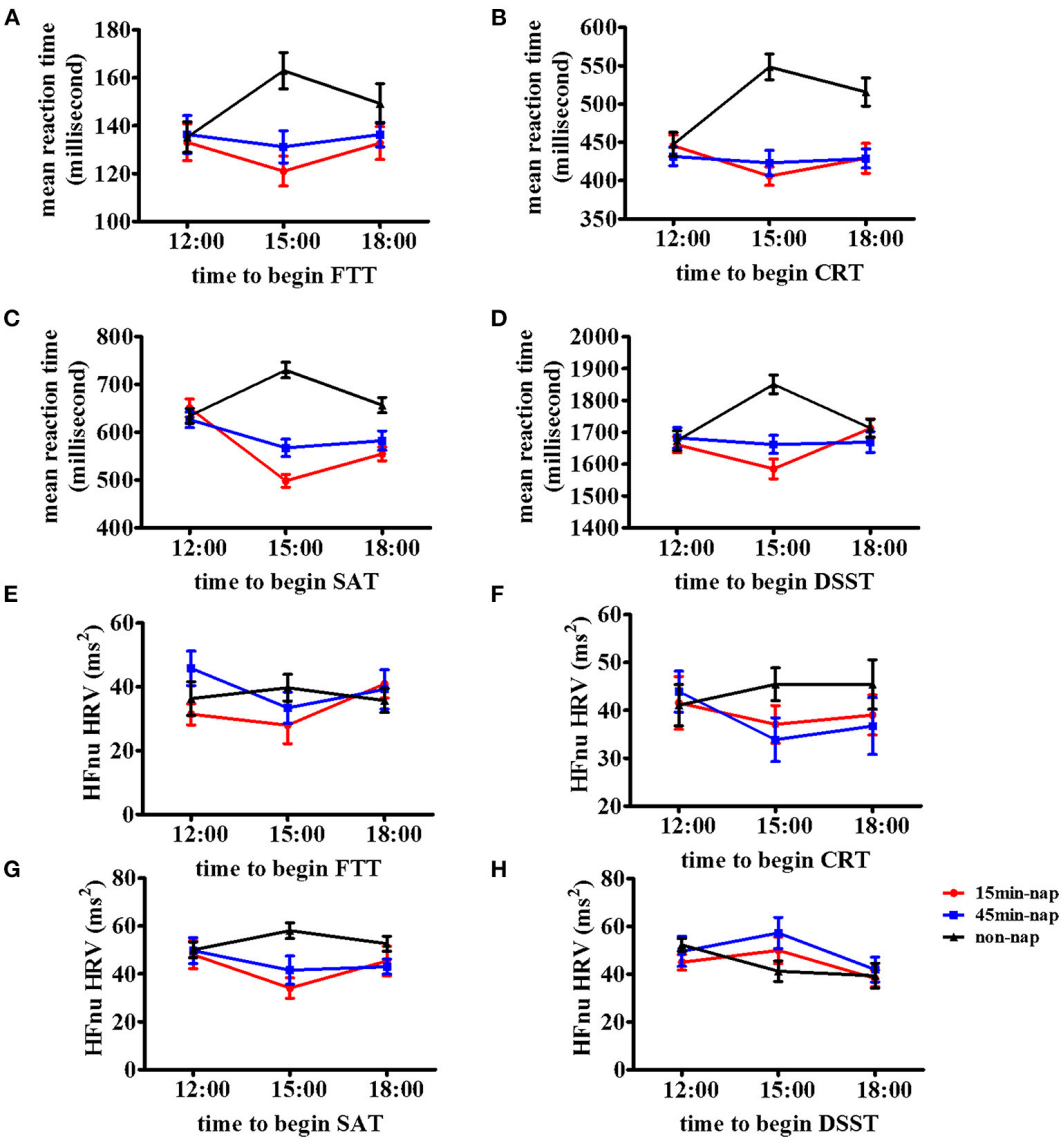
Mean and stand deviations of cognitive tests and resting vmHRV of the time course in the three nap groups. Means and standard deviations are shown for four outcome metrics of the cognitive tests and resting vmHRV among three nap groups (non-nap, 15-min nap, and 45-min nap). Time course of the **(A)** FTT performance, **(B)** CRT performance, **(C)** SAT performance, **(D)** DSST performance, **(E)** HFnu (FTT), **(F)** HFnu (CRT), **(G)** HFnu (SAT), and **(H)** HFnu (DSST) in participants under nonnap group (lines with triangle), 15-min nap group (lines with square), or 45-min nap group (lines with circle).

Figure 2 shows the changes in reaction time from the 12:00 baseline to the 15:00 and 18:00 times for all nap groups for the measures of (A) FTT, (B) CRT, (C) SAT, (D) DSST, (E) HFnu (FTT), (F) HFnu (CRT), (G) HFnu (SAT), and (H) HFnu (DSST). This method of illustration provides the relative changes following different nap durations from 12:00 to both 15:00 and 18:00. Figure 2 shows the change in the mean reaction time in the cognitive tasks across all nap groups over time. It presents the general increase in reaction time in the non-nap group and a decrease in reaction time following 15-min and 45-min naps, with a regression back toward the non-nap group at 18:00 after both the 15-min and 45-min naps.

Resting vmHRV analyses revealed significant planned contrasts of the interaction effects between the non-nap group and 15-min nap group were HFnu (SAT) at 15:00 and HFnu (FTT) and HFnu (DSST) at 18:00. In the 45-min nap group, the only significant planned contrast of the interaction effects was HFnu (DSST) at 18:00.

### *Post hoc* examination of nap conditions

Improvements in cognitive performance were evident following the 15-min and 45-min naps in this study. These improvements are graphically indicated in Figure 2 as a relatively reduced reaction time after the nap at 15:00 and subsequently increased values. *Post hoc* 2-way repeated-measures ANOVAs were performed, with 2 levels of the factor nap group (non-nap and 15-min nap) and 3 levels of the factor time (12:00, 15:00 and 18:00). In the event of a significant interaction effect, simple within-subjects planned contrasts were then performed to compare the non-nap and 15-min nap groups between the 12:00 baseline and the 2 subsequent test times, with Bonferroni adjustments due to the exploratory nature of this analysis. As presented in Table 3, significant interactions between the non-nap and 15-min nap groups were observed between 12:00 and 15:00 for all cognitive outcome variables and between 12:00 and 18:00 for FTT and CRT performance. The same analysis for the non-nap vs. 45-min nap comparison also found significant interaction effects across the three test times. The interaction between the 2 nap groups (non-nap and 45-min nap) and time (12:00 and 15:00) approached significance for all cognitive outcome measures. A significant interaction between the 2 nap groups (non-nap and 45-min nap) and time (12:00 and 18:00) was found for FTT and CRT performance.

**Table 3:** Two-way ANOVA and planned within-subjects contrasts for condition (non-nap vs. 15-min nap) by time for the dependent variables.

**Variable**	**ANOVA**	**Condition**	**Planned contrasts**	
			**Time**	
	**F**		**F**	
			**12:00 (baseline)** **vs. 15:00**	**12:00 (baseline)** **vs. 18:00**
FTT	21.74***	Non-nap vs. 15-min nap	42.73***	9.33**
CRT	29.14***	Non-nap vs. 15-min nap	50.89***	30.62***
SAT	16.05***	Non-nap vs. 15-min nap	16.25***	3.00
DSST	4.86**	Non-nap vs. 15-min nap	5.33*	0.01
HFnu(FTT)	5.49**	Non-nap vs. 15-min nap	8.97**	1.24
HFnu(CRT)	0.16	Non-nap vs. 15-min nap	–	–
HFnu(SAT)	7.69***	Non-nap vs. 15-min nap	8.02**	0.57
HFnu(DSST)	7.34**	Non-nap vs. 15-min nap	15.12***	0.35

ANOVA refers to analysis of variance. FTT, Finger Tapping Test; CRT, Choice Reaction Time; DSST, Digit Symbol Substitution Test; and SAT, Shifting Attention Test; HFnu, high-frequency normalized units. * *p* < 0.05, ** *p* < 0.01, *** *p* < 0.001.

Similarly, repeated-measures ANOVA of resting vmHRV revealed a significant main effect of HFnu (FTT), HFnu (SAT) and HFnu (DSST), with 2 levels of the factor nap group (non-nap and 15-min nap) and 3 levels of the factor time (12:00, 15:00 and 18:00). In the event of a significant interaction effect, planned contrasts were performed to compare the interaction effects of changes in the non-nap and 15-min nap groups between 12:00 and each post-nap testing period, with Bonferroni-adjusted criteria. Significant interactions between the non-nap and 15-min nap groups were observed between 12:00 and 15:00 for FTT, SAT and DSST performance. In addition, as shown in Table 4, the analysis of the non-nap vs. 45-min nap comparison found significant interaction effects across the three test times. Significant interactions between the non-nap and 45-min nap groups were observed only between 12:00 and 15:00 for HFnu (DSST).

**Table 4:** Two-way ANOVA and planned within-subjects contrasts for time (pre-nap vs. post-nap time) by condition (non-nap vs. 45-min nap) for the dependent variables.

**Variable**	**ANOVA**	**Condition**	**Planned contrasts**	
			**Time**	
	**F**		**F**	
			**12:00 (baseline)** **vs. 15:00**	**12:00 (baseline)** **vs. 18:00**
FTT	20.63***	Non-nap vs. 45-min nap	41.36***	6.27*
CRT	20.01***	Non-nap vs. 45-min nap	47.41***	16.04***
SAT	3.80*	Non-nap vs. 45-min nap	10.25**	1.16
DSST	4.47*	Non-nap vs. 45-min nap	4.46*	0.01
HFnu(FTT)	2.59	Non-nap vs. 45-min nap	–	–
HFnu(CRT)	0.67	Non-nap vs. 45-min nap	–	–
HFnu(SAT)	4.67*	Non-nap vs. 45-min nap	2.62	1.48
HFnu(DSST)	5.72*	Non-nap vs. 45-min nap	10.76**	0.01

ANOVA refers to analysis of variance. FTT, Finger Tapping Test; CRT, Choice Reaction Time; DSST, Digit Symbol Substitution Test; and SAT, Shifting Attention Test; HFnu, high-frequency normalized units. * *p* < 0.05, ** *p* < 0.01, *** *p* < 0.001.

### Comparison of the 15 and 45-min nap conditions

The relative benefits of cognitive performance following the 15 and 45-min naps were compared with *post hoc* analyses. For all outcome measures, 2-way repeated-measures ANOVAs were performed with the factors nap group (15-min nap and 45-min nap) and time (12:00, 15:00, and 18:00). In the event of an overall significant interaction term, planned contrasts were performed to compare the simple interaction effect of changes in the 15 and 45-min nap groups between 12:00 and each post-nap testing period, with Bonferroni-adjusted criteria. As shown in Table 5, the 15-min nap produced significantly greater benefits at 15:00 after napping than did the 45-min nap for FTT performance. While all the other comparisons up until 18:00 tended to favor the performance of the 15-min nap group, none of these interaction effects were statistically significant. The same analysis on resting vmHRV after the 15-min and 45-min naps showed no significant interaction effects across the three test sessions.

**Table 5:** Two-way ANOVA and planned within-subjects contrasts for time (pre-nap vs. post-nap time) by condition (15-min vs. 45-min) for the dependent variables.

**Variable**	**ANOVA**	**Condition**	**Planned contrasts**	
			**Time**	
	**F**		**F**	
			**12:00 (baseline)** **vs. 15:00**	**12:00 (baseline)** **vs. 18:00**
FTT	6.38*	15-min nap vs. 45-min nap	8.91**	2.51
CRT	2.99	15-min nap vs. 45-min nap	–	–
SAT	0.78	15-min nap vs. 45-min nap	–	–
DSST	1.30	15-min nap vs. 45-min nap	–	–
HFnu(FTT)	1.13	15-min nap vs. 45-min nap	–	–
HFnu(CRT)	1.10	15-min nap vs. 45-min nap	–	–
HFnu(SAT)	0.28	15-min nap vs. 45-min nap	–	–
HFnu(DSST)	0.11	15-min nap vs. 45-min nap	–	–

ANOVA refers to analysis of variance. FTT, Finger Tapping Test; CRT, Choice Reaction Time; DSST, Digit Symbol Substitution Test; and SAT, Shifting Attention Test; HFnu, high-frequency normalized units. * *p* < 0.05, ** *p* < 0.01, *** *p* < 0.001.

### Regression analyses with parasympathetic activity during cognitive tasks

Next, the study evaluated the importance of resting vmHRV for cognitive performance across different nap durations at 12:00 (baseline), 15:00, and 18:00 with hierarchical linear regression. Two linear regression models were built to predict resting vmHRV variables for FTT, CRT, SAT, and DSST performance. The method of analysis was to estimate a separate hierarchical linear regression for different cognitive tasks due to the differences in independent variables. Regarding FTT performance, Model 1 included FTT performance across the three nap groups for the three test sessions. In Model 2, we added HFnu during FTT performance. Model 1 was significant, *F*_(3, 101)_ = 26.27, *p* < 0.001, adjusted R^2^ = 0.47, which demonstrated that different nap lengths had a strong impact on FTT performance in the three test sessions. There was no significantly predicted performance in Model 2 [*F*_(6, 98)_ = 14.61, *p* = 0.12, adjusted R^2^ = 0.52]. Regarding CRT performance, Model 1 included CRT performance across the three nap groups for the three test sessions. In Model 2, we added HFnu during CRT performance. Model 1 was significant [F_(3, 101)_ = 26.53, *p* < 0.001, adjusted R^2^ = 0.52], which suggested that different nap lengths had a strong impact on CRT performance in the three test sessions. There was no significantly predicted performance in Model 2 [*F*_(6, 98)_ = 13.00, *p* = 0.83, adjusted R^2^ = 0.50]. The same analysis of predictive resting vmHRV variables regarding SAT performance revealed that Model 1 was significant [*F*_(3, 101)_ = 9.25, *p* < 0.001, adjusted R^2^ = 0.26], which demonstrated that different nap lengths had a strong impact on SAT performance in the three test sessions. Model 2 also significantly predicted performance, F_(6, 98)_ = 10.69, *p* < 0.001, adjusted R^2^ = 0.45, R^2^ = 19.1%), with HFnu as a significant predictor (*p* < 0.001). Furthermore, the analysis of predictive resting vmHRV variables regarding DSST performance showed that Model 1 was significant [*F*_(3, 101)_ = 22.08, *p* < 0.001, adjusted R^2^ = 0.46], which suggested that different nap conditions had a strong impact on DSST performance in the three test sessions. Model 2 also significantly predicted performance [*F*_(6, 98)_ = 13.76, *p* = 0.03, adjusted R^2^ = 0.51, R^2^ = 6.5%], with HFnu as a significant predictor (*p* < 0.001).

### Regression analyses between DSST performance and other cognitive tasks

To identify predictors of DSST performance, regression analyses were undertaken. The effect of the predictor variables would not be identical across the three nap groups at the three times. Thus, the method of analysis was to estimate a separate regression for each group to identify indicator variables and the key predictor variables. Variables such as motor speed, relational selection, and attentional shift influenced DSST performance across the three nap groups. In the 15-min nap group, the multiple *R* value for the equation was *R* = 0.32 (*p* = 0.48) at 12:00. After the 15-min nap (15:00), the regression equation included FTT (beta = 5.98; *p* < 0.001), CRT (beta = 0.09; *p* = 0.24), and SAT (beta = 0.18; *p* = 0.55) scores. The multiple *R* value for the equation was *R* = 0.71 (*p* < 0.001). At 18:00 in the 15-min nap group, the regression equation included FTT (beta = 4.79; *p* < 0.001), CRT (beta = 0.02; *p* = 0.76), and SAT (beta = 0.04; *p* = 0.22) scores. The multiple *R* value for the equation was *R* = 0.724 (*p* < 0.01). In the other groups, there were no significant effects related to DSST performance. In the 45-min nap group, the multiple *R* values for the equation were *R* = 0.18 (*p* = 0.87) at 12:00, *R* = 0.42 (*p* = 0.12) at 15:00, and *R* = 0.15 (*p* = 0.91) at 18:00. In the non-nap group, the multiple *R* values for the equation were *R* = 0.42 (*p* = 0.12) at 12:00, *R* = 0.13 (*p* = 0.93) at 15:00, and *R* = 0.20 at 18:00 (*p* = 0.81).

## Discussion

This study provides insights into the effects of different nap durations associated with cognitive performance and HRV in interns. The participants were interns after a nightshift. We investigated the consequences on cognitive demands and fluctuations in autonomic activity after differing durations of daytime naps in interns. We present evidence that naps as brief as 15 min are considered one of the best strategies for sleep management, especially among interns, and are an effective method for improving information processing, motor speed, response selection, and attention shifting. Resting vmHRV during cognitive task performance was associated with SAT and DSST scores. Our results provide evidence of a potential role for parasympathetic activity during cognitive task performance in interns.

Based on the study, interns can limit fatigue and improve cognitive performance through naps. Strategies to improve circadian adaptation and relieve the stress include exogenous melatonin, hypnotics after work, bright light exposure, and virtual reality (VR). Melatonin (at a dose 1.8 to 3.0 mg) promote sleep quality during the day after night shifts (47). Although the effect of melatonin in improve circadian alignment has been confirmed (48), the chronic use and healthy safety for interns need further research. Hypnotics including temazepam (49), triazolam (50), and zolpidem (51) taken after night shift could improve sleep quality not cognitive performance. As one adaptation strategy, research has found that bright light exposure facilitates circadian adaptation, mood, and cognitive performance during the night shift (52, 53). Light exposure between 2500 to 10000 lux ranged from 5 sections of 15 min each to 6 h (52, 54), but the improvement of performance in daytime was not seen (55). Chaojin Chen (55) provided evidence to confirm the use of virtual reality (VR) technology to reduce stress among anesthesiologists during night shifts. The limitation of using VR technology was another extra qualified anesthesiologist arranged to take over the subjects' work during the VR immersion to make sure the safety of patients. However, it may not achieve because extra anesthesiologists are not always present. Therefore, daytime napping could be one effective and impractical strategy to improve circadian adaptation for interns.

Previous studies have shown an inverted U-shaped association between napping lengths and cognitive performance (56). For example, non-nap and long-nap groups (90 min) were associated with worse cognitive performance, and moderate nap lengths were associated with better cognitive performance (56). Omar Boukhris and colleagues found that a 90-min daytime nap opportunity was better than 40 min for attention and negative mood states in fourteen healthy adults (57). Compared with a non-nap group, the results of a previous study revealed that a 30-min nap significantly improved alertness and cognitive performance in thirteen healthy males (58). However, 4 weeks of nap restriction or practice was not effective in achieving better performance in either group, such that restricting naps in nap+ participants (who napped at least once a week) did not reduce the cognitive benefits they derived from naps and increasing naps in nap- individuals (who rarely or never napped) did not enhance nap-related perceptual learning (59)^.^ The contradictions among these results could be related to the nap lengths and individual differences. Our findings support and extend results from studies with cognitive tests, such as the DSST, CRT, SAT, and FTT, carried out in non-nap, 15 and 45-min nap groups. In this study, cognitive performance was significantly improved after 15-min naps and 45-min naps. Cognitive performance was significantly decreased in the non-nap group. It is noteworthy that more beneficial effects were observed after a 15-min nap than after a 45-min nap, which suggested that 15-min naps were associated with better cognitive performance.

This study examined the impact of different nap durations on cognitive performance in the context of autonomic activity. Previous studies on resting vmHRV provided support for the theoretical and neuroimaging links between vmHRV and cognitive task performance (60). Behavioral evidence has shown that higher resting vmHRV predicted better performance in a wide range of cognitive tasks (61). Similarly, research has demonstrated that vmHRV was also associated with executive function—a recent meta-analysis of functional magnetic resonance imaging (fMRI) investigations showed that higher resting vmHRV was associated with better performance on a battery of tasks involving executive function (34). To test this model, researchers have proposed that the relationships between vmHRV during the task and cognitive performance are specific to the task. In this sense, better task performance is linked to decreased or increased vmHRV depending on the characteristics of different tasks (62). For example, high resting vmHRV was associated with better performance in executive function and working memory tasks than low resting HRV, whereas there were no differences in simple tasks. High resting vmHRV is also associated with goal-oriented tasks that require more self-regulatory effort (62–64). Individuals with higher resting vmHRV showed a more adaptive vagal response in a given situation (62, 65). These reports support that the varying levels of resting vmHRV are associated with in-task vmHRV and task performance, such that it could index the degree to which executive brain areas are flexible depending on task demands (65). Nevertheless, the differences observed in the present study among the resting vmHRV values across the cognitive tasks appear unusual based on the traditional interpretation. Based on the traditional interpretation, the extent of our study examined the differences among the resting vmHRV values in relation to the cognitive tasks following different nap durations. We found that HFnu suppressed the resting vmHRV in the FTT and CRT, and HFnu increased stimulation of the resting vmHRV in the DSST and SAT in 15-min nap and 45-min nap groups with faster mean reaction times. Meanwhile, HFnu showed suppression of the resting vmHRV in the DSST and SAT and increased the stimulation of the resting vmHRV in the FTT and CRT in the non-nap group with slower mean reaction times. Our findings indicated an increased stimulation of sympathetic and vagus systems depending on the different cognitive components that were engaged. The findings agree with Hansen (45), who suggested that a high resting vmHRV group showed better performance, including reaction time and accuracy parameters in executive tasks. They also showed that cognitive tests can alter HRV, and the effects on high resting vmHRV were specific to executive tasks (66). Other studies have also indicated that behavior, which is sensitive to executive function, during the tests leads to an increase in the resting vmHRV (28, 29). According to different cognitive components associated with executive and non-executive function, the results indicated that participants with high resting vmHRV had faster mean reaction times on executive tasks such as the DSST and SAT, whereas those with low resting vmHRV had faster mean reaction times on non-executive tasks including the FTT and CRT; these findings suggested that the FTT and CRT are less related to prefrontal activation than the DSST and SAT.

In addition, our study provided evidence for the cognitive mechanisms underlying DSST performance across the different nap duration conditions. DSST performance recruits different interrelated abilities, such as motor-based skills, response selection (67), and shifting of attention (68). We performed regression analyses to evaluate the contribution of the different components to DSST performance. In previous studies, motor speed was considered a component of the DSST measured by the FTT and showed significant impairments in an Asperger disorder (AD) group (15), while motor speed was negligibly affected in the normal group (69). According to the regression analysis in our study, FTT was significantly relevant to DSST performance across the different nap durations in the three time sessions, which suggested that motor speed was an important factor influencing DSST performance. Response selection measured by the CRT made little contribution to DSST performance. Traditional cognitive components that are considered critical for determining good performance in the DSST are motor speed, relational selection, and attentional shift. We found that attentional shift measured by the SAT had no significant contribution to DSST performance. However, this finding was inconsistent with a previous study (68), which showed that attentional shift is one of the significant predictors of DSST performance. We speculate that nap durations influence this process, especially the adverse effect of non-naps on DSST performance. Furthermore, successful DSST performance does not depend only on motor speed, response selection and attentional shift, but there is evidence that this process may also require working memory and visual scanning efficiency (70). Working memory and visual scanning efficiency appear essential to DSST performance. Working memory has an important influence on attentional shift, which has supervisory functions, such as error monitoring and activating task-appropriate schema (71). Visual scanning efficiency indicates that participants consult the code key frequently during the test administration (72). The principal scanning operations in the performance of the DSST involve at least two saccades: from the test item to the code key and from the code key to the test item, which suggests that appropriate saccadic eye movements do not rely on memory.

## Conclusion

Studies of the effects of different nap durations on cognitive performance in the context of autonomic nervous system (ANS) activity are few. This study demonstrated that a brief nap can serve as a countermeasure against cognitive impairment and that autonomic activity while awake played a crucial role in DSST and SAT performance across the different nap durations. Motor speed was a significant predictor of DSST performance in relation to the effects of daytime napping. Furthermore, the unexplored domains of the central nervous system and the relationship of the ANS with cognitive performance, which promotes associative cognitive processes during napping, should be considered in future studies. In addition, considering that taking brief naps has critical practical implications, daytime napping has potential benefits for hospital staff.

## Data availability statement

The original contributions presented in the study are included in the article/supplementary material, further inquiries can be directed to the corresponding author/s.

## Ethics statement

The studies involving human participants were reviewed and approved by First Affiliated Hospital of Air Force Medical University (KY20213076-1). The patients/participants provided their written informed consent to participate in this study.

## Author contributions

Conceptualization: LW and XS. Methodology, formal analysis, and writing—original draft preparation: JF and LW. Software: XY and XCZ. Validation: JF, YW, and XS. Investigation: JF, LW, ZS, SW, LZ, XL, XBZ, and CL. Data curation: JF, LW, YP, YT, and XS. Writing—review and editing: JF and ZL. Visualization: LW and ZL. Project administration: LW and YW. Funding acquisition: YW, ZL, and XS. Resources: ZL. Supervision: LW and XY. All authors have read and agreed to the published version of the manuscript.

## Funding

This research was supported by grants from National Natural Science Foundation of China: Grant No. 82003038, Key R&D Program of Shaanxi Province: Grant No. 2021SF-020, 2021SF-426, and 2020JM-317.

## Conflict of interest

The authors declare that the research was conducted in the absence of any commercial or financial relationships that could be construed as a potential conflict of interest.

## Publisher's note

All claims expressed in this article are solely those of the authors and do not necessarily represent those of their affiliated organizations, or those of the publisher, the editors and the reviewers. Any product that may be evaluated in this article, or claim that may be made by its manufacturer, is not guaranteed or endorsed by the publisher.
